# Charge Air System in an Experimental Combustion Engine—Combined Simulation Model: A Digital Twin Approach Including Advanced Control Concepts

**DOI:** 10.3390/s26123854

**Published:** 2026-06-17

**Authors:** Miki Sirola, Jaber McBreen, Mohammad Raisi Esfarjani

**Affiliations:** 1 Energy Technology, University of Vaasa, 65101 Vaasa, Finland; jaber.mcbreen@uwasa.fi (J.M.); mohammad.raisi.esfarjani@uwasa.fi (M.R.E.); 2 Environmental Soil Science, Department of Agricultural Sciences, Faculty of Agriculture and Forestry, Institute of Atmospheric and Earth System Research, University of Helsinki, P.O. Box 56, FI-00014 Helsinki, Finland

**Keywords:** digital twin, simulation model, charge air system, experimental combustion engine, advanced control

## Abstract

The larger research problem is to get combustion engines more effective and flexible and reduce or even eliminate greenhouse gas emissions. Here we concentrate more on a smaller-scale and focused research problem about the significance of air feeding in engine operation. Therefore, the need for modeling a charge air system is obvious. The interaction and co-operation between the charge air systems and combustion engines is a central issue in this article. A literature review was carried out on related topics, and it reveals a research gap in this area. A simulation model of a charge air system based on first principles is developed. It is based on physical and systemic modeling, and it is constructed including control loops reducing and controlling the pressures in the charge air chain. The simulation models of this auxiliary system and engine are successfully combined, and functioning together is demonstrated. The composed models represent real research laboratory equipment in the University of Vaasa Energy Laboratory under construction. The research laboratory equipment and the whole research environment are described. Simulation scenarios are presented both with the charge air system alone and with the combined model, including also the engine part. The significance of the developed models is discussed, and the path towards a digital twin experiment environment is outlined. As a conclusion, we can claim that the combined simulation model is successfully constructed and shown to operate in a stable and physically plausible manner. The digital twin concept can be tested completely only when the research laboratory is constructed and ready and the test runs begin to produce measurement data for the digital part. Then also the simulation models can be tuned to a better accuracy level, and the operation as a digital twin will be verified.

## 1. Introduction

The increasing demand for higher efficiency, lower emissions, and improved operational flexibility in internal combustion engines has accelerated the development of advanced modeling and control methodologies. Among these, digital twin technology has emerged as a powerful paradigm, enabling the integration of real-time data with physics-based simulation models to replicate and predict system behavior under varying operating conditions. Digital twins are increasingly applied in energy systems and engine research to enhance diagnostics, optimization, and control performance [1,2,3].

The charge air system plays a critical role in the performance and efficiency of combustion engines. It directly influences air mass flow, pressure, temperature, and humidity—parameters that strongly affect combustion quality, emissions formation, and overall engine efficiency [4,5]. Accurate modeling of the charge air process is therefore essential, especially in modern research environments where flexible operation and alternative fuels are investigated. However, the complex interactions between thermodynamics, fluid dynamics, and control mechanisms present significant challenges for both modeling and real-time implementation.

To address these challenges, component-oriented modeling approaches based on first principles have been widely adopted. Such approaches decompose the system into physically meaningful subsystems such as compressors, tanks, valves, heat exchangers, and control loops, allowing detailed representation of individual processes while maintaining computational tractability [6,7,8]. In the present work, the charge air system is modeled as a chain of interconnected components, capturing the dynamic evolution of pressure, temperature, mass flow, and humidity throughout the system. The model incorporates thermodynamic relations, moist air theory, and simplified empirical relations where appropriate, enabling a balance between physical fidelity and simulation efficiency.

In addition to physical modeling, control strategies are essential for maintaining stable and desired operating conditions. Pressure regulation via feedback control loops is commonly used in air handling systems, where valve positions are adjusted to maintain target pressures under varying load conditions [9]. In this study, two pressure-based control loops are implemented to regulate system behavior, reflecting practical industrial control concepts while ensuring numerical robustness and stability.

The research is conducted within a newly developed experimental infrastructure at the University of Vaasa, where a single-cylinder research combustion engine is integrated with multiple auxiliary systems, including the charge air system. This setup provides a flexible platform for studying advanced combustion concepts and air management strategies. The charge air system itself consists of multiple process components such as compressors, tanks, dryers, filters, heaters, and coolers, forming a complex and highly configurable air supply chain. The integration of a simulation-based digital twin with this physical test bench enables detailed analysis of system dynamics and supports the development of advanced control and optimization strategies.

At the current stage, the framework primarily represents a physics-based virtual counterpart of the future laboratory setup. A fully operational digital twin additionally requires continuous real-time synchronization, bidirectional data exchange, online calibration, and adaptive updating using measurements from the physical system. These functionalities remain future work after commissioning of the experimental infrastructure. Because the laboratory system is still under construction, the present study should be interpreted mainly as a proof-of-concept integrated modeling framework rather than a fully validated operational digital twin.

Despite extensive research in engine modeling and air system control, there remains a gap in integrated digital twin implementations that combine detailed component-level modeling with experimental research platforms. Many existing studies focus either on high-fidelity computational models with limited real-time applicability or simplified control-oriented models lacking physical interpretability. Existing studies typically focus either on conceptual digital twin architectures, isolated engine models, or simplified air path simulations, while fewer studies integrate component-level charge air system modeling, humidity handling, pressure control strategies, and experimental engine-platform coupling within a unified simulation framework. This work aims to bridge this gap by developing a physically grounded yet computationally efficient digital twin of a charge air system, tightly coupled with an experimental engine setup.

It should be emphasized that the present work represents a preliminary simulation-based stage toward a future digital twin implementation. Because the physical laboratory setup is still under construction, experimental validation and calibration using measured data are not yet possible. Consequently, the presented results should be interpreted primarily as proof-of-concept demonstrations of the integrated modeling and control framework rather than fully validated predictive simulations.

In addition to subsystem model integration, the developed framework is intended to support future research on fault diagnosis, virtual commissioning, supervisory control, and operational optimization of the research engine platform.

The main contributions of this paper are as follows:

- Development of a component-based simulation model of a charge air system using first principles and empirical formulations.
- Integration of moist air thermodynamics and condensation effects within a system-level model.
- Implementation of practical control loops for pressure regulation.
- Coupling of the simulation model with a real experimental research engine environment, forming a digital twin framework.

This paper is structured as follows: Section 2 reviews related work on charge air system modeling and digital twins. Section 3 presents the experimental test bench. Section 4 describes the simulation model in detail. Section 5 describes the engine model and the integration of the charge air system as one example of the auxiliary systems to it. In Section 6 simulation results are presented, followed by discussion and conclusions.

## 2. Related Work

Digital twin technology has received growing attention in recent years as a framework for linking physical assets with virtual models for monitoring, diagnosis, prediction, and control. Broader reviews in manufacturing, transportation, and automotive applications consistently describe digital twins as a combination of a physical system, a virtual counterpart, data exchange, and application-layer services such as optimization or maintenance support [3,10,11,12]. In automotive and power-system contexts, the literature also emphasizes that the practical value of a digital twin depends not only on model fidelity but also on computational efficiency, sensor integration, and the ability to update the model with measured operating data [10,11,12].

Within internal-combustion-engine research, digital twins have been proposed for performance monitoring, health assessment, calibration support, and emissions-related studies. Recent reviews specifically focused on internal combustion engines note that the field is moving from purely offline simulation toward hybrid frameworks in which physics-based models are coupled with measured signals and, in some cases, data-driven corrections [12,13]. This trend is consistent with the wider engine-modeling literature, where the need for real-time capable models has long motivated the development of reduced-order and control-oriented representations rather than full high-fidelity computational fluid dynamics models [6,7,14]. For experimental engine platforms, this distinction is especially important because the model must remain physically interpretable while being fast enough for controller development, hardware-in-the-loop studies, or online supervisory functions [13,14,15].

The previous literature also emphasizes the importance of validation using experimental measurements and quantitative performance metrics such as transient tracking accuracy, pressure error statistics, settling times, and controller response characteristics. However, such validation stages are strongly dependent on the availability of fully instrumented experimental facilities. In the present work, extensive experimental validation remains future work because the physical laboratory system is still under construction.

A substantial body of prior work has addressed control-oriented engine and air path modeling. Established monographs and review papers by Guzzella and Onder, Eriksson and Nielsen, and later combustion-modeling reviews show that lumped-parameter and mean-value approaches remain central for representing engine subsystems with acceptable computational cost [6,7,15]. These approaches typically describe the dynamics of pressure, temperature, and mass flow through manifolds, throttles, compressors, and actuators using conservation laws and simplified constitutive relations. Their main advantage is that they preserve physical structure and parameter meaning while remaining suitable for simulation-based control design [6,7]. Similar ideas have also been used in simplified intake-system and whole-engine models, especially when the research objective is system integration rather than detailed local flow resolution [16].

Regarding the charge air and intake path specifically, prior studies have examined thermodynamic conditioning of air intake, pressure losses, and the effect of air path states on engine behavior. Earlier thermodynamic analyses of charge air conditioning systems demonstrated that intake-air temperature and pressure management can significantly influence air density and engine operating characteristics [17]. More recent studies have extended this perspective by examining condensation, icing, and humidity-related phenomena in intake systems, particularly under challenging ambient or recirculated gas conditions [18,19]. These results are relevant because they show that moisture behavior cannot always be neglected in air path studies, especially when cooling, drying, or humidification processes are present [18,19].

The role of intake or charge air humidity has also been studied from the viewpoint of combustion and emissions. Experimental and numerical investigations have shown that humidity changes can influence combustion temperature, oxygen availability, and nitrogen oxide formation, while also interacting with intake temperature and turbocharging constraints [20,21,22,23]. Serrano et al., for example, reported significant changes in engine-out NOx when charge air humidity was varied under altitude-related operating conditions [20]. Other studies similarly indicate that humidification or ambient-humidity variation may alter thermal efficiency, heat-transfer behavior, and pollutant formation [21,22,23]. These findings support the inclusion of humidity-related states in system-level models when the target application involves realistic air preparation rather than dry-air-only assumptions.

In parallel, researchers have continued to improve component-level subsystem models for compressors, tanks, valves, and thermal devices. In practical engineering environments, such models are often built as lumped or semi-empirical modules representing the dominant physics of each component while neglecting small-scale internal phenomena [6,7,16]. This modular philosophy is particularly attractive for digital twin development because it facilitates model maintenance, calibration, and extension when new hardware is added to a test bench. It also supports the combination of first-principles descriptions for the most influential dynamics with empirical relations for secondary effects, thereby providing a reasonable compromise between fidelity and robustness [7,11,13].

Despite these advances, the literature still shows a gap between three partially separate streams of work:

1. Digital twin studies that emphasize architecture, monitoring, or data integration but provide only limited physical detail for the air path.
2. Engine air path and control-oriented models that are physically meaningful but are not framed as digital twins connected to an experimental platform; and
3. Humidity- or condensation-related studies that focus on a single phenomenon without integrating the entire charge air preparation chain [6,7,13,14,18,19,20,21,22,23]. For the present research context, this gap is important because the target system is not only an engine model but a complete experimental charge air system consisting of compressors, tanks, dryers, filters, valves, heaters, coolers, branching flow paths, and pressure control loops connected to a research engine platform.

The contribution of the present work is therefore positioned at the intersection of these streams. Unlike purely conceptual digital twin studies, the proposed framework is built around an explicit component-based simulation model of the charge air system. Unlike many conventional air path models, it also incorporates humidity handling and drying/cooling effects as part of the system-level formulation. In addition, the model is intended for integration with a real experimental research environment, which is a key requirement for a practical digital twin but is less commonly demonstrated in the literature on charge air subsystem modeling. This combination makes the work relevant both to engine-system simulation and to sensor-informed digital twin development for laboratory-scale combustion-engine research.

Nevertheless, the present work should still be considered a preliminary digital twin-oriented framework because closed-loop synchronization and experimental calibration with the physical laboratory infrastructure are not yet available.

## 3. Research Test Bench in Energy Laboratory

Engine Laboratory 3 as a part of Vaasa University’s energy laboratory is being built during the year 2026. The becoming research infrastructure using the acronym SIRU consists of a one-cylinder research combustion engine and four auxiliary systems connected to the engine.

The research engine is a single-cylinder, four-stroke laboratory engine derived conceptually from the cylinder geometry of a known Finnish diesel engine model. It uses the same bore and stroke proportions as one cylinder of the original 5 L four-cylinder engine, resulting in a displacement of approximately 1.25 L. The engine is water-cooled and equipped with a four-valve cylinder head adapted for research purposes. The design allows installation of multiple pressure and temperature sensors, and the cylinder head can optionally accommodate optical access for advanced combustion diagnostics.

The engine is designed primarily for controlled laboratory testing and combustion research. A robust crankshaft with large counterweights and a balance shaft is used to reduce the inherent vibration typical of single-cylinder engines. Fuel delivery is based on a high-pressure common rail injection system, initially configured for diesel fuel but designed to be modular so that alternative fuels can later be introduced. The intake system can use either turbocharging or an external laboratory compressor to precisely control boost pressure and air supply.

In its initial diesel configuration, the engine would have an estimated displacement of about 1.25 L, a maximum operating speed of approximately 2000–2500 rpm, and a peak output in the range of 40 kW (@1900 RPM), with maximum torque roughly 240 Nm (@1400 RPM) depending on boost and fueling settings. The combustion system is designed to tolerate peak cylinder pressures up to roughly 175–200 bar, allowing the study of modern and future combustion concepts. The mechanical durability limit is around 250–300 bar. The modular design makes it suitable for later experiments with alternative fuels such as synthetic fuels, alcohols, gaseous fuels, or hydrogen.

**Figure 1:**
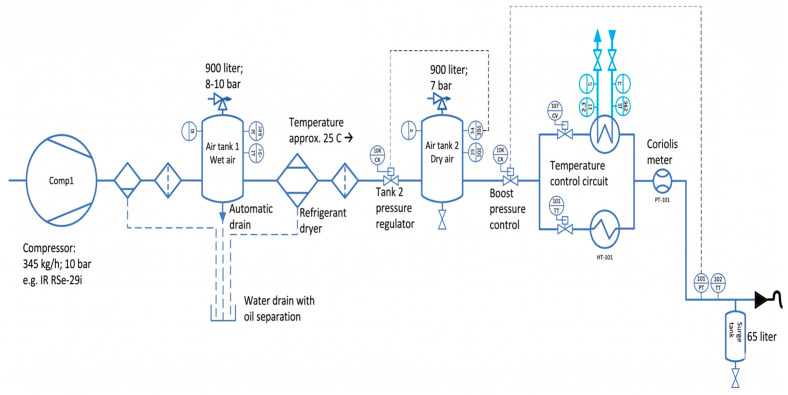
Charge air system in the SIRU energy laboratory.

The SIRU engine has four main auxiliary systems: fuel (diesel in the beginning) feeding system, cooling system, charge air system, and exhaust system. In addition, an engine brake called a dynamometer is connected to the engine shaft. The charge air system, as seen in Figure 1, is described here in more detail than the other systems because a simulation model of it is a focus area in this article.

The air charge system constitutes a chain of process components preparing suitable air feed to the engine in terms of pressure, temperature, humidity and mass flow. The process components are a compressor, two air tanks, two dryers, two filters, a cooler, a heater, and control valves. The positions of two valves are controlled by traditional PID controllers. This article discusses a digital twin of this charge air system and its connection to an engine simulation model.

## 4. Simulation Model of Charge Air System for Research Engine

A simulation model of a charge air system feeding air for a research engine in a real university research test bench is composed. The model is built in the Simulink platform using mostly MATLAB (R2025b) functions representing process components in a chain. The model is built by using first principles and physical laws component by component. Pure Simulink components are needed as well.

The idea in the model is to describe the dynamic behavior of mass flow, pressure, temperature, and humidity throughout the system and the interactions between these quantities in different operating conditions. The system consists of a sequence of functional components such as compressors, filters, dryers, tanks, valves, and heat exchangers, as well as a branched flow path that later recombines. The air flow progresses logically from the inlet toward the engine, and the structure of the model follows this physical order. The system includes two control loops used to regulate valves based on measured pressures. The control loops share the same structural principle, and their purpose is to stabilize system operation under varying conditions.

The compressor model is based on thermodynamics and moist air theory [24]. The main physical principles are the ideal gas model for dry air, isentropic compression with efficiency correction, energy balance, moist air partial pressure and saturation theory. If the relative humidity at the outlet exceeds 100%, the excess water vapor is assumed to condense.

The compressor power is calculated from the air enthalpy increase,

(1) $$\dot{W}=\dot{m}\;c_{p}\left(T_{\mathrm{out}}-T_{\mathrm{in}}\right)$$

where power is a function of mass flow, temperature difference and specific heat capacity at constant pressure. Water vapor is modeled using Dalton’s law [24,25] of partial pressures and a temperature-dependent saturation pressure calculated with the Magnus approximation [26].

The tank model is based on mass and energy conservation for compressible gas in a fixed volume [24]. The tank pressure follows the ideal gas law [24], while mass and internal energy change due to inflow, outflow through an orifice, pressure relief venting, and heat exchange with the surroundings. Flow through the outlet and relief valve is driven by pressure differences and modeled using orifice flow relations, with smooth activation to avoid discontinuities.

The general form of tank pressure calculation is written,

(2) $$\frac{dp}{dt}=\frac{RT}{V}({\dot{m}}_{in}-{\dot{m}}_{out})$$

where p is the tank pressure, R is the specific gas constant of air, T is the air temperature, V is the tank volume and is the mass flow of incoming and outgoing air.

The dryers and filters are modeled in a simpler manner with empirical, static and linear models. The dryer model is a representation of moisture removal (drying) and heat transfer. The filter model represents pressure loss in a flow restriction [24].

The combined cooler and dryer model represents a combined cooling and drying process, where air temperature is actively reduced toward a target range and moisture is removed as a function of the cooling intensity. Moisture removal is assumed to increase with stronger cooling, reflecting condensation-based drying behavior. The process causes a small reduction in mass flow due to water removal [27].

Wet air condensation calculation is presented in formula

(3) $$q_{cond}=q_{da}\;(w_{in}-w_{sat})$$

where q_cond_ is the condensate mass flow rate, q_da_ is the dry air mass flow rate, w_in_ is the inlet humidity ratio, and w_sat_ is the saturation humidity ratio.

The valve model represents a flow-restricting control valve whose effective opening determines the transmitted mass flow and downstream pressure. By adjusting its opening position, the valve introduces a controllable flow restriction that regulates both mass flow and pressure in the downstream line. The model includes actuator dynamics, a deadband, and a nonlinear flow characteristic to represent typical real valve behavior. As the valve opening increases, the flow capacity increases in a nonlinear manner, reflecting the characteristic relationship between valve position and flow in practical control valves [28].

The control loop is based on feedback pressure control, where the tank pressure is regulated by adjusting the upstream valve opening. The objective of control is to maintain the measured pressure at a specified set point by modulating the mass flow into the tank [29]. More details regarding the control loops are shown in Section Pressure Control Strategy.

In the splitter, the total mass flow is divided into two branches based on their relative flow resistances. Physically, this corresponds to flow distribution in parallel channels, where a larger effective opening (lower flow resistance) results in a larger share of the total mass flow being directed to that branch [24].

The cooler represents a heat exchanger in which the air flow transfers heat to a colder cooling medium. Physically, the air temperature approaches the coolant temperature according to the effectiveness of the heat transfer process. In this simplified model, the pressure drop across the cooler is assumed to be negligible [24].

The heater represents a component in which the temperature of the air flow is increased using an external heat source. Physically, the air temperature approaches the temperature of the heating source according to the effectiveness of the heat transfer process. The mass flow and pressure are assumed to remain unchanged as the air passes through the heater [24].

The mixer represents the merging of two air flows into a single combined flow. Physically, the total mass flow of the merged stream is the sum of the branch mass flows, and the resulting temperature is determined from an energy balance as a mass-flow-weighted average of the inlet temperatures. The pressure at the mixing point is approximated by the lower of the two branch pressures, representing a simplified limitation imposed by the branch with lower pressure [24].

After the mixer, no new physical phenomena are introduced in the model. The purpose of the remaining part of the chain is to collect, route, and present the quantities supplied to the engine. The mixer provides three primary quantities: pressure, temperature, and mass flow. In addition, air humidity is taken from an upstream location where it was last modified and is assumed to remain constant for the remainder of the system.

In addition, the model includes needed sources and sinks. At the end of the chain just before the warm air is fed to the engine, there is a small ‘breathing tank’ in the real-world test setting. This is graphically represented in the model but has no effect on the calculation simulation results.

**Figure 2:**
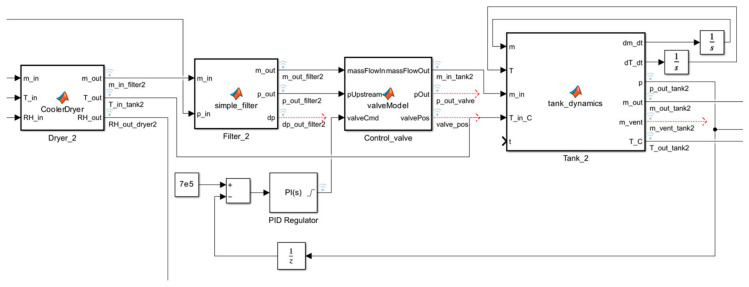
A sample part from the charge air simulation model.

Note that the pipes are not modeled in this simulation model. The changes in pressure, temperature, etc., are assumed to be small enough to keep the model entity somewhat plausible by ignoring this part. In Figure 2 a snapshot of part of the Simulink model is seen.

The simulation model is built and tuned according to the best practices to give the most plausible results possible in this phase of the work, considering that we do not have any comparison data yet. We have used the best expertise of our laboratory to estimate how the system will operate when it is constructed and ready. The time for accurate verification and validation will come later when real measurement data exists.

### Pressure Control Strategy

To ensure stable operation of the charge air system under transient and load-dependent conditions, pressure regulation is achieved using two proportional–integral (PI) controllers acting on flow control valves. The controllers regulate pressures at two physically distinct locations along the air preparation chain: an intermediate storage tank and the system outlet feeding the engine. This decentralized pressure control structure follows established industrial practice for gas and air handling systems and offers robustness with low computational complexity [6,7,9].

PI control was selected because of its simplicity, robustness, low computational demand, and widespread industrial use in pressure-regulation applications.

In both control loops, the controlled variable is a measured pressure signal, and the manipulated variable is the corresponding valve opening position. The control error is defined as the difference between the pressure setpoint and the measured pressure. Each PI controller is implemented in continuous time as

(4) $$u\left(t\right)=K_{p}e\left(t\right)+K_{i}\int_{0}^{t}e(\tau )d\tau$$

where $K_{p}$ and $K_{i}$ denote the proportional and integral gains, respectively. The controller output is mapped to a normalized valve position, which modifies the effective flow area and thereby regulates the downstream pressure through a controllable flow restriction.

One controller stabilizes the pressure level within the charge air system, acting as a buffer against upstream disturbances such as compressor dynamics and mass-flow variations. The second controller regulates the final delivery pressure supplied to the engine, compensating for disturbances caused by upstream tank dynamics, flow splitting, and changes in engine air demand when the engine model is coupled. Similar pressure-based control concepts are widely used in control-oriented intake and air path models for internal combustion engines [6,7,15].

The two PI controllers operate on different subsystems and time scales. Because the outlet pressure depends indirectly on upstream tank dynamics and flow distribution, the two pressure control loops are dynamically coupled. However, the coupling remained sufficiently weak to allow sequential controller tuning and stable operation during the simulated startup scenario. The controller parameters were tuned iteratively using simulation-based trial-and-error testing. The proportional and integral gains were adjusted sequentially to obtain stable pressure responses with acceptable settling behavior and limited oscillation during startup transients. The upstream pressure loop was tuned first because its dynamics strongly influence downstream pressure behavior. During tuning, larger proportional gains produced faster responses but increased oscillatory behavior, while smaller gains improved damping at the expense of slower pressure stabilization. The final parameter values were selected as a compromise between response speed, numerical stability, and oscillation magnitude.

Integral action ensures zero steady state pressure error, while derivative action is omitted to improve numerical robustness and reduce sensitivity to pressure signal noise. This choice is particularly relevant for a digital twin-oriented framework intended for later integration with real sensor data [9,11]. The tuned parameters used in the simulations are summarized in Table 1.

The controller implementation assumes ideal pressure measurements without sensor noise or communication delay because the present study focuses on preliminary simulation-model development. The PI controllers are implemented in continuous time within the Simulink environment using a simulation sample time of 0.01 s. Actuator saturation, anti-windup mechanisms, and measurement filtering are not included at this stage. These implementation details will be extended later when experimental instrumentation and measured data become available from the physical laboratory system.

## 5. Engine Model and Model Coupling

**Figure 3:**
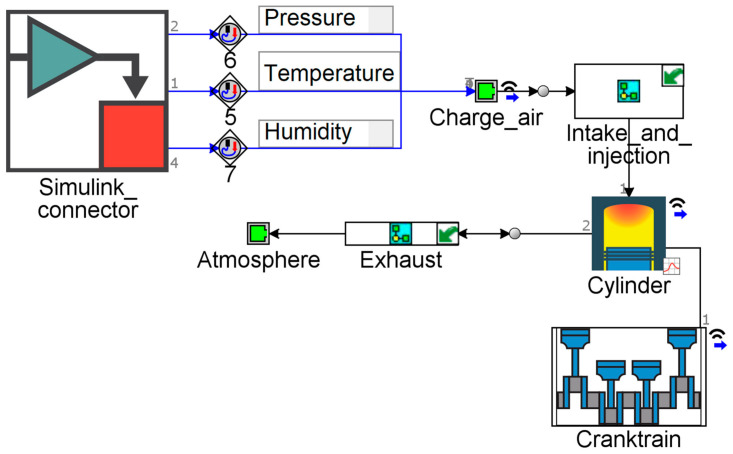
A placeholder single-cylinder engine model.

To pre-emptively emulate the upcoming laboratory setting, an engine model is coupled to the simulation. As the ultimate goal will be to hold a true digital twin of the laboratory setting, including both the charge air system and a validated engine model, the same methods expected to be applied later in the process were used to include a representative engine model. The tool used for engine simulation is GT-Power v2024 (Gamma Technologies, Westmont, IL, USA) [30], a simulation tool that allows true one-dimensional flow simulation along the engine’s air path. In the absence of internal engine dimensional data, it is assumed that the engine is a conventionally operated single-cylinder SI engine with a displacement of 1.25 L. The modeling approach is particularly targeted for system-level simulations, and only functions vital to assessing the efficacy of this coupling method are kept along at this stage. A model overview can be found in Figure 3.

As Simulink is used as the governing simulation tool, the engine model is interfaced with it through a coupling object, which directly imposes the temperature, pressure and relative humidity of the compressor output at the intake runner. As the engine will be modified to operate in SI mode, the injection controller targets a stoichiometric air-fuel ratio throughout the simulation. As a performance-oriented model, the engine is set to run at a constant user-imposed speed, thus waiving inertia-induced effects for the sake of simulation simplicity and speed.

## 6. Simulation Results

An application example is described to demonstrate the basic functionalities of the charge air system simulation model. This scenario is a cold startup of a charge air system. Normally, and especially in stand-by state, the two main tanks in a charge air system are pressurized, which makes the startup of this auxiliary system much faster. Here, we simulate a kind of first startup, which is done in the beginning of taking the system in use or after a longer maintenance break. The starting point from initial conditions is when, in both 900 L tanks, there is 1 bar pressure, normal room temperature and humidity, and no mass flow, making a delay of many minutes before the steady state of the system is reached.

**Figure 4:**
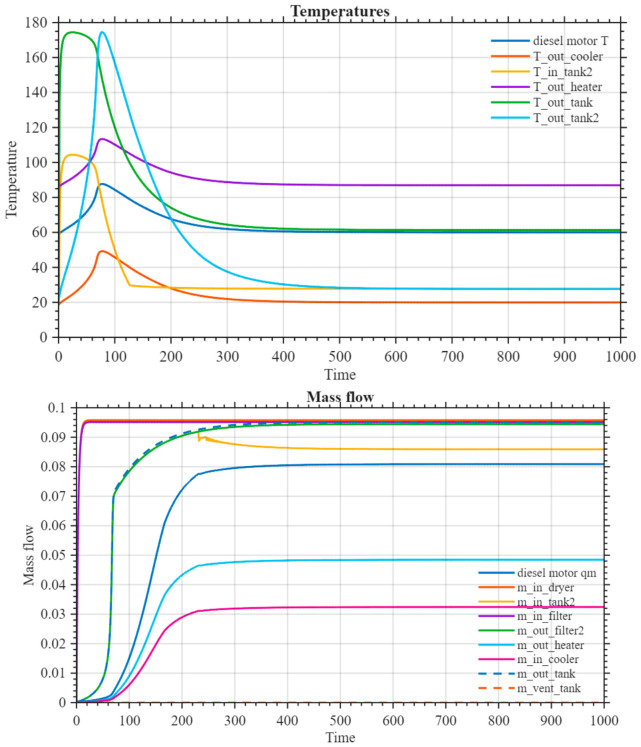

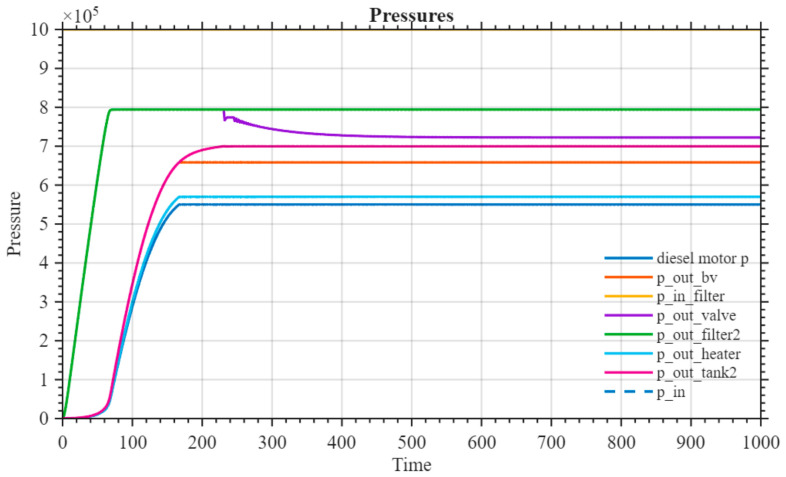
Temperature, pressure and mass flow in different parts of the charge air chain during the scenario.

In Figure 4 temperatures, pressures and mass flows in different parts of the charge air chain are seen. During the startup transient, temperatures in both big tanks rise first rather high before the steady state is reached. The whole simulation run takes 1000 s (16.67 min). At the end of the scenario, the temperature of the air going into the diesel engine is 60 degrees Celsius, the pressure is 5.5 bar, the mass flow is 0.081 kg/s and the relative humidity is 8.7%. The charge air chain pressurizes, heats up and dries the air when preparing it for the engine.

Pressures for the second tank in the charge air chain and engine feeding are controlled with two separate control loop valves acting as actuators. Pressures are measured in the second tank and just before feeding the air to the engine in the charge air chain. The values are compared to the setpoints, and two PI controllers take care of adjusting the valve positions so that the setpoint values for the pressures are reached.

The simulation results indicate that both pressure control loops operate in a stable manner during the startup transient. The intermediate tank-pressure loop reaches the vicinity of its pressure setpoint within 171 s with only minor overshoot (Table 2). The outlet pressure loop exhibits slower dynamics and moderate oscillatory behavior caused by coupling between the two pressure control loops and varying mass-flow conditions in the branched air path. However, the oscillations remain bound and gradually decay toward stable operation. The responses obtained demonstrate that the implemented PI controllers are capable of maintaining physically plausible and stable pressure regulation under transient operating conditions.

A description of the engine model operation follows. In the combined model where the two different models are combined, the charge air system as an auxiliary system and the engine model itself operate together. The main variables on the engine side follow logically the behavior of the auxiliary system connected to it. The most essential variables to follow on the engine side are the power of the engine and controlled fuel consumption.

**Figure 5:**
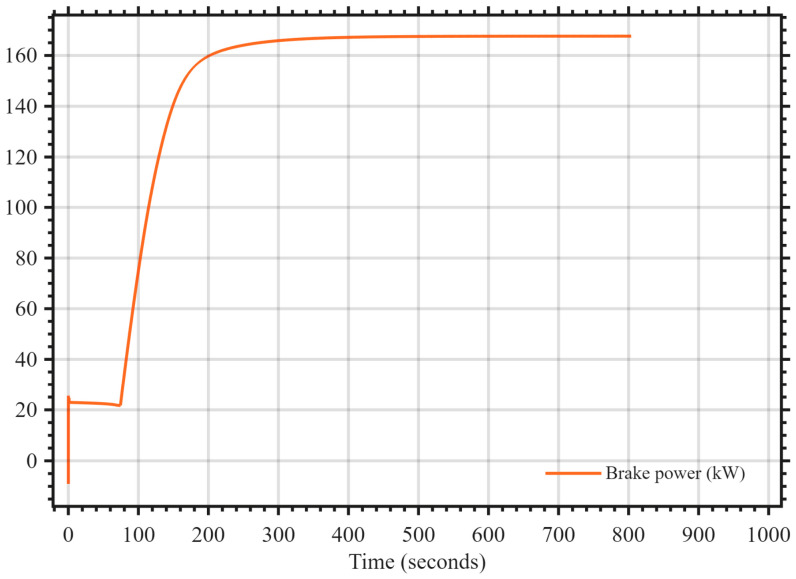
Brake power response to changing intake pressure.

**Figure 6:**
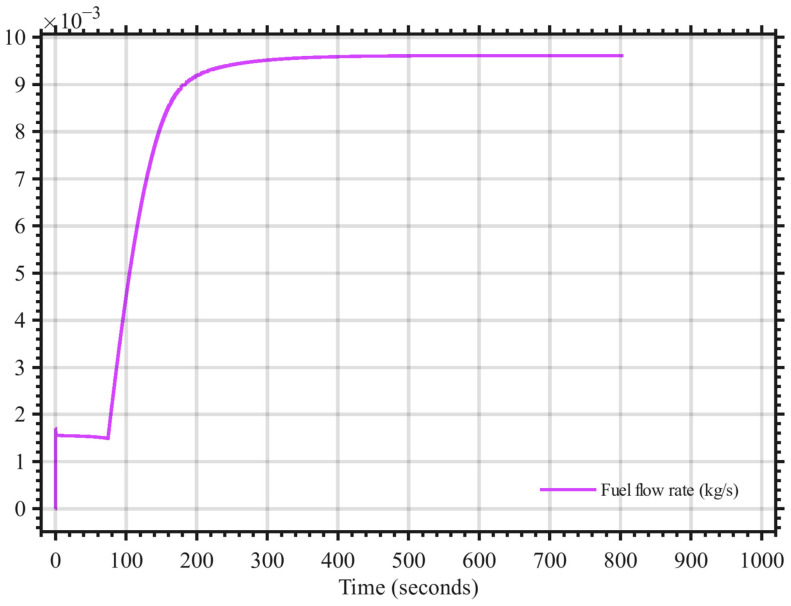
Fuel consumption response to changing intake pressure.

The engine model accepts the signals for pressure, temperature and relative humidity and imposes them at the air intake, and the injection controller adjusts fuel injection accordingly to maintain the stoichiometric air-fuel ratio. Thus, the brake power and fuel flow rate follow closely the charge air pressure curve, as is seen in Figure 5 and Figure 6.

## 7. Discussion

We have built an experimental combined simulation model of a charge air system connected to a one-cylinder experimental diesel engine to show how these two separate systems can operate together on a system-level simulation. The charge air system has been modeled in the Simulink environment using mostly MATLAB functions as components, and the engine model has been modeled using GT Power. These two models in different environments have been technically successfully combined to operate together.

This experimental setup is being built during this year, 2026, in Engine Laboratory 3 as a part of the energy lab at the University of Vaasa. When the laboratory equipment is operating in real life too, we will combine our simulation model combination with data received from the real system to build a digital twin concept out of it according to our approach in this article.

The simulation models heading towards the digital twin concept are in a development phase and not fully finalized yet. There are still many possibilities for technical improvements as well as ground for such research goals as emission reduction. There are many possibilities, including thinking of the role of the charge air system in this respect.

Our charge air system is built as a physical model based on first principles. The model is not accurate yet, but it can already help in planning and developing a real system, including planning and development of control systems both in the charge air system and on the motor side, as these two models are connected.

The model has been developed component by component and put in a chain, just as they are composed in the corresponding real system. Note that the pipes themselves have not been modeled in this rough model. The pressure losses, etc., in pipes are assumed to be small enough compared to the changes in modeled components. This must be considered in the tuning of the model in an increasingly realistic direction.

In our description of the model, we have written out only a couple of the most important equations. To write here more equations could increase the assertiveness of our model, but on the other hand they are all based on very basic physical principles and can be easily found from the literature that we have referenced. Therefore, we have decided not to put every possible equation connected to this model visible.

A limited qualitative sensitivity analysis was performed during controller tuning by testing multiple proportional and integral gain combinations in simulation. Increasing proportional gains generally improved response speed but produced stronger oscillatory behavior in the coupled pressure control loops. Lower controller gains improved damping but significantly increased settling time. The selected controller parameters were therefore chosen as a compromise between stability, acceptable transient response, and oscillation reduction under startup conditions.

We try to fill in a research gap between combining models of input air path to a diesel motor to the engine itself, which can be later operated with other fuels as well, giving fewer emissions, and finally also with hydrogen solutions even without any emissions. We have made contributions to the component-wise charge air system simulation model, integrating thermohydraulic principles in it and controlling pressures with traditional control loops. Combining a charge air model and an engine model successfully is a key role in our work.

We have glanced through the literature of digital twin technology, internal combustion engine research, control-oriented engine and air path modeling, charge air intake path, thermohydraulic conditioning of intake air, etc. In suck air we concentrate on following four important physical variables: temperature, pressure, mass flow and humidity.

We have noticed some gaps in the literature, especially in such things as limited physical detail for air paths, digital twin frames in experimental platforms, humidity and condensation, and air paths component by component, and try to fill these to some extent.

We have described and modeled a real laboratory system, which is currently in the planning phase, and described at some level the modeling details and technical solutions in the realization process. The ready system will open many more possibilities by means of, e.g., accuracy.

We show the simulation results of the charge air system alone and, combined with the engine model, show how the engine model reacts to the input from an auxiliary system and discuss challenges and problems with controlling this kind of system entity.

The present study has several limitations that should be acknowledged. The simulation model has not yet been validated against experimental measurements because the physical laboratory setup is still under construction. In addition, several practical effects such as sensor noise, actuator saturation, pipe pressure losses, transport delays, and measurement uncertainty are not yet included in the model. The implemented PI controllers were tuned using simulation-based trial-and-error methods and have not yet been optimized systematically. Future work will focus on experimental calibration, validation against measured data, and extension toward a fully synchronized digital twin environment.

## 8. Conclusions

A combined simulation model including a charge air system as an auxiliary system and a one-cylinder research engine model has been composed. The two models are built in completely different computing environments: the charge air system model in Simulink/MATLAB and the engine model in GT Power. Both models represent a real technical system that is currently being built in motor laboratory 3 in the energy lab of the University of Vaasa. This kind of combination is unique, and we did not find any similar systems with similar features from the literature. Technically combining these two models is described in Section 5.

The charge air system simulation model is built from chained components, each based on physical principles. The physical models include integration of thermohydraulic phenomena, energy balance, mass balance, enthalpy, conservation of energy, ideal gas behavior, etc., basic physical principles. The model based on 1st principles is described in more detail in Section 4. The control loops controlling pressures in the charge air system are built and tuned according to basic principles from control theory [29].

The next step is to complete these simulation models to reach digital twin status when the real laboratory equipment is in its place and functioning well enough. The idea is then to use the measurement data from the research laboratory environment to tune these existing models with much better accuracy than they can have at this stage. Now our simulation examples show that these two models operate together and produce already plausible output depending functionally on each other.

## Figures and Tables

**Table 1:** Tuned parameters of the PI pressure controllers used in the charge air system simulation.

Control Loop Location	Controlled Variable	Manipulated Variable	$K_{p}$	$K_{i}$	Pressure Setpoint [Pa]
Intermediate pressure loop	Tank pressure	Control valve opening	0.01	0.0001	700,000
Outlet pressure loop	Mixer outlet pressure	Boost valve opening	0.001	0.0001	550,000

**Table 2:** Dynamic performance characteristics of the implemented pressure control loops.

Control Loop	Pressure Setpoint [Bar]	Steady-State Error [Bar]	Settling Time [s]	Overshoot
Intermediate tank pressure	7	0.0008	171	No Overshoot
Outlet pressure loop	5.5	0.001	153	No Overshoot

## Data Availability

The raw data supporting the conclusions of this article will be made available by the authors on request.
